# A51 INTESTINAL MICROBIOTA DETERMINES ARYL HYDROCARBON RECEPTOR ACTIVATION AND SUSCEPTIBILITY TO COLITIS

**DOI:** 10.1093/jcag/gwac036.051

**Published:** 2023-03-07

**Authors:** L Rondeau, D Haas, X Wang, A Caminero

**Affiliations:** Medicine, McMaster University, Hamilton, ON, Canada

## Abstract

**Background:**

Intestinal microbiota, diet, and the immune system have been proposed to contribute to the development of inflammatory bowel diseases (IBD). The aryl hydrocarbon receptor (AhR) is a critical regulator of intestinal immunity and mucosal barrier homeostasis that is activated by agonists such as host and microbial tryptophan metabolites. IBD patients have altered microbiota and reduced AhR agonists in intestinal content resulting in the downregulation of AhR. These findings necessitate further study to understand how diet-microbiota interactions contribute to intestinal inflammation.

**Purpose:**

To study the influence of intestinal microbiota on tryptophan metabolism, AhR activation, and colitis severity.

**Method:**

8- to 10-week-old germ-free C57BL/6 mice were colonized with cecal content of mice harbouring specific pathogen-free (SPF) or a limited and well-defined altered Schaedler flora (ASF) microbiota. Germ-free mice were used as controls. Three weeks following colonization, mucosal injury was induced in a subset of mice with dextran sulfate sodium (DSS) in drinking water for five days followed by two days of water recovery. Activation of AhR was measured in stool using an *in vitro* AhR luciferase reporter assay. Stool AhR agonists were determined using high-performance liquid chromatography coupled to high-resolution mass spectrometry. Colonic expression of AhR pathway genes *Cyp1a1*, *Il22*, *Ahrr, Ahr*, and *Il17* was evaluated by RT-qPCR. Susceptibility to colitis was assessed by analysing stool consistency and stool blood, colonic microscopic damage, immune infiltration by immunohistochemistry, and pro-inflammatory gene expression (NanoString). Fecal microbiota was analysed by 16S rRNA gene sequencing (Illumina).

**Result(s):**

AhR agonists, AhR activation *in vitro*, and AhR pathway gene expression were elevated in mice colonized with SPF microbiota in comparison to mice colonized with ASF microbiota and germ-free mice. In ASF-colonized mice, DSS induced more severe inflammation than in SPF-colonized mice, as demonstrated by worsened mucosal injury, greater weight loss, and softer stools. SPF-colonized mice developed less mucosal immune cell infiltration and pro-inflammatory gene signaling.

**Conclusion(s):**

Our findings suggest that intestinal microbiota composition determines the metabolic capacity to degrade tryptophan into agonists that homeostatically activate AhR. When mucosal injury is induced, mice with elevated microbiota-derived AhR agonists and AhR activation develop less severe mucosal injury and signs of colitis. This study presents a useful tool for evaluating dietary and microbial therapies in the context of a microbiota with impaired tryptophan metabolism.

**Please acknowledge all funding agencies by checking the applicable boxes below:**

CCC, CIHR

**Disclosure of Interest:**

None Declared

